# Silencing of Long Non-coding RNA MIAT Sensitizes Lung Cancer Cells to Gefitinib by Epigenetically Regulating miR-34a

**DOI:** 10.3389/fphar.2018.00082

**Published:** 2018-02-13

**Authors:** Yunfeng Fu, Chengyuan Li, Yanwei Luo, Lian Li, Jing Liu, Rong Gui

**Affiliations:** The Third Xiangya Hospital of Central South University, Changsha, China

**Keywords:** long non-coding RNA, lung cancer, epigenetic, acquired resistance, c-Met

## Abstract

Long non-coding RNA (lncRNA) myocardial infarction associated transcript (MIAT) was recently identified as oncogene in several cancers. However, the role of MIAT on acquired resistance in lung cancer and the underlying mechanisms remain unclear. Here, we showed that the expression of MIAT in lung cancer tissues was upregulated compared with adjacent tissues. LncRNA MIAT expression was associated with tumor size, lymph node metastasis, distant metastasis and TNM stage. Univariate analysis and multivariate analysis revealed that the lncRNA MIAT to be an independent factor for predicating the prognosis of lung cancer patients. Low lncRNA MIAT have longer overall survival time and progression-free survival time than patients with high lncRNA MIAT expression. Moreover, the knockdown of MIAT significantly sensitized PC9 and gefitinib-resistant PC9 cells to gefitinib *in vitro* and *in vivo*, and increased the expression of miR-34a and inactivated PI3K/Akt signaling. MIAT interacted with miR-34a and epigenetically controlled the miR-34a expression by hyper-methylating its promotor. Taken together, our findings demonstrated that knockdown of MIAT by siRNA enhances lung cancer cells to gefitinib through the PI3K/Akt signaling pathway by epigenetically regulating miR-34a. Thus, MIAT may be a useful prognostic marker and therapeutic target for lung cancer patients.

## Introduction

Lung cancer, including small-cell lung cancer (SCLC, accounting for about 20%) and non-small-cell lung cancer (NSCLC, accounting for about 80%), is a leading cause of cancer deaths worldwide. Amount of genomic and epigenomic alterations have been revealed during the development of lung cancer (Ma et al., 2017). The NSCLC cases often harbor epidermal growth factor receptor (EGFR) activating mutation. EGFR-tyrosine kinase inhibitors (EGFR-TKIs) are the first line chemotherapy drugs for EGFR-mutant NSCLC patients. However, acquired drug resistance often occurs on the continuous treatment (Zhang and Yuan, 2016). Once activated by ligands and receptor dimerization, EGFR can activate cellular signaling pathways such as the phosphoinositide 3-kinase (PI3K)-AKT pathway, ultimately leading to increased cell proliferation and survival. The mutations or genetic polymorphisms of EGFR may result in primary resistance on EGFR-TKIs, and gene copy alterations in pathways frequently induce acquired resistance, such as MET is considered one of the more common causes of acquired resistance in EGFR-mutant NSCLC (Rotow and Bivona, 2017). In addition, the epigenetic mechanisms are also indicated to be an important pathway for acquired TKI resistance (Stewart et al., 2015).

Long non-coding RNAs (lncRNAs) are a group of non-coding RNAs those length greater than 200 nucleotides. LncRNAs exhibit their functions through multiple biological mechanisms involving epigenetic, transcriptional, and post-transcriptional alterations (Chen Y. et al., 2016). Dysfunctions of lncRNAs are associated with tumorigenesis of various cancers, including lung cancer (Tian et al., 2017). Emerging evidence has revealed that lncRNAs play essential roles in drug resistance of cancer, such as glioma, bladder cancer, breast cancer and lung cancer (Niu et al., 2017). Li H. et al. (2017) revealed that lncRNA MALAT1 decreased the sensitivity of glioma cells to temozolomide by regulating ZEB1. LncRNA MEG3 overexpression induced cisplatin sensitivity of NSCLC cells by regulating miR-21-5p/SOX7 axis (Wang et al., 2017). LncRNA AK001796 increased the resistance of NSCLC cells to cisplatin through regulating cell apoptosis and cell proliferation (Liu et al., 2017). LncRNAs may serve as potential diagnostic biomarkers or therapeutic targets in lung cancer (Gong et al., 2017; Xue et al., 2017).

LncRNA MIAT was originally identified in myocardial infarction (MI) on chromosome 22q12 that confers risk of MI (Ishii et al., 2006). The following studies found that lncRNA MIAT was involved in various diseases, such as MI, diabetic retinopathy, paranoid schizophrenia, microvascular dysfunction and cancer (Liao J. et al., 2016; Zhou et al., 2017). LncRNA MIAT promoted breast cancer progression by regulating miR-155-5p to regulate DUSP7 expression in breast cancer (Luan et al., 2017), and promoted proliferation and invasion of hepatocellular carcinoma cells via regulating miR-214 (Huang et al., 2017). And Zhang HY showed that knockdown of MIAT substantially inhibited the invasive ability of NSCLC cells through the ZEB1 signaling pathway by regulating miR-150 (Zhang et al., 2017). However, whether MIAT plays a role on drug resistance in lung cancer remain unknown.

In this study, we focus on delineating role of lncRNA MIAT on drug resistance in lung cancer. We investigated the expression of MIAT in lung cancer tissues, and the association between MIAT and prognosis of lung cancer patients. Furthermore, we have analyzed role of MIAT in gefitinib resistance by employing genetic manipulation techniques. Our findings provide evidence that inhibition of MIAT may overcome EGFR-TKI acquired resistance in lung cancer.

## Materials and Methods

### Study Population

Two hundred and twelve lung cancer tissues were collected from non-small cell lung cancer patients with mutant EGFR between February and April 2011. The patients who had prolonged stable disease of more than 6 months or a partial response and complete response to EGFR-TKIs therapy were defined as sensitive patients. The 2009 patients with progression on the first imaging evaluation or stable disease less than 6 months after EGFR-TKI treatment in the first setting were defined as primary resistance to EGFR-TKI. There are also 186 cases of adjacent tissue as a control. The present study was approved by the Ethics Committee of the Third Xiangya Hospital of Central South University. Written informed consent was obtained from the participants of this study.

### Cell Culture

The human normal pulmonary epithelial cell BEAS-2B and the human lung cancer cell lines PC9, HCC827, A549, H1870, H1048, and H1299U87 were obtained from Chinese Academy of Medical Sciences (Beijing, China). Cells were cultured with Dulbecco’s modified Eagle’s medium (DMEM) containing 10% fetal bovine serum (FBS, Gibco-BRL) and maintained in a humidified atmosphere of 5% CO_2_ in air at 37°C.

The gefitinib-resistant PC9 cell line (PC9/R) was established in our lab. The PC9 cells (EGFR mutations on exon 19; 1 × 10^5^/ml) were incubated for 24 h, and then treated with the initial concentration of gefitinib (0.5 μM). The medium containing gefitinib was changed once every 2–3 days. When the initial dose was induced for 2 weeks, the drug dose was doubled, and each dose was maintained for 2 weeks. The final concentration was increased to 20 μM.

### Reagents

The following antibodies were used in this study: GAPDH (Santa Cruz Biotechnology, Dallas, TX, United States), Bcl-2, Bax, cleaved caspase 3, c-Met, p-EGFR(pY1068), EGFR, p-PI3K(pY607), PI3K, p-Akt(pS473), Akt, Dnmt3a, Dnmt3b, and Dnmt1 (Abcam, United Kingdom). Gefitinib (cat no. SML1657) was obtained from Sigma–Aldrich.

### Cell Treatment

The packaged lentivirus containing MIAT shRNA and empty vector control were purchased from Genepharma Company (Beijing, China). The PC9 and PC9/R cells were infected with these lentiviruses. Briefly, the cells (2 × 10^5^ cells per well) were seeded in 6-well plates and cultured until they reached 80–90% confluence. Then the cells were infected with lentivirus at multiplicity of infection (MOI) of 50 in the presence of 5 mg/ml polybrene (Sigma–Aldrich, St. Louis, MO, United States) for 48 h.

Inhibition of miR-34a was performed using miR-34a inhibitor (GeneCopoecia, Guangzhou, China). The cells transfected with inhibitor control were used as negative control. Cells were plated in 6-well plates or 96-well plates and transfected for 24 h or 48 h by using Lipofectamine 3000 (Invitrogen) according to the manufacturer’s instructions. Briefly, PC9 and PC9/R cells (2 × 10^5^ cells per well) were seeded separately in 6-well plates and cultured until they reached 80–90% confluence. Then, the cells were transfected with miR-34a inhibitors in serum- and antibiotic-free medium for 6 h. Afterward, the medium was refreshed, and the cells were continuously cultured for 48 h.

### RNA Extraction and SYBR Green Quantitative PCR Analysis

Total RNA was extracted from cells using Trizol reagent (Invitrogen, Carlsbad, CA, United States). Total RNA was reverse transcribed using a transcription kit (Invitrogen) to get total cDNA as templates for MIAT detection, or transcribed with Takara^TM^ microRNA transcription kit to get cDNA as templates for miR-34a detection. The expression of MIAT was measured by SYBR green qPCR assay (Takara, Dalian, China) according to manufacturers’ instructions. Expression of β-actin was used as an endogenous control. MiR-34a expression was detected using a Hairpin-it TM miRNAs qPCR kit (Genepharma, Shanghai, China) according to manufacturers’ instructions. Expression of RNU6B was used as an endogenous control. QPCR was performed at the condition: 95.0°C for 3 min, and 39 circles of 95.0°C for 10 s and 60°C for 30 s. Data were processed using 2^-ΔΔCT^ method. The primers were used as following: 5′- TCCCATTCCCGGAAGCTAGA -3′(forward), 5′- GAGGCATGAAATCACCCCCA -3′(reverse) for MIAT. 5′- TTGTTACAGGAAGTCCCTTGCC-3′(forward), 5′- ATGCTATCACCTCCCCTGTGTG-3′(reverse) for β-actin. Sequence of miRNA and references using in current study are: miR-34a: UGGCAGUGUCUUAGCUGGUUGU; RNU6B: CGCAAGGAUGACACGCAAAUUCGUGAAGCGUUCCAUAUUUUU. The reverse primers were also used in the Hairpin-it TM miRNAs qPCR kit.

### Cell Viability Assay and Colony Formation Assay

Twenty-four hours before the experiment, the cells were plated in 96-well plates at a density of 1000 cells in 100 μl medium per well. The cells were treated with a range of gefitinib, and the cell viability was assessed by CCK-8 assay (Beyotime, Shanghai, China) according to the manufacturers’ instructions.

For the colony formation assay, following treatment, adherent cells were trypsinized and 1000 viable cells were subcultured in six-well plates (in triplicate). Cells were allowed to adhere and cultured for 2 weeks in absence or presence of gefitinib as indicated. To visualize colonies, media were removed and cells were fixed in 96% ethanol for 10min, and then stained with 0.5% crystal violet (Beyotime) for 30 min. The colonies were captured and counted.

### Flow Cytometry for Apoptosis Analysis

Staining was performed by using Annexin V-FITC kit (KeyGEN BioTech, Nanjing, China) following the manufacturer’s instructions. Briefly, 2 × 10^5^ PC9 or PC9/R cells were harvested by centrifugation at 1000 × *g* for 5 min and resuspended in 100 μl binding buffer. The cells were incubated with 5 μl Annexin V-FITC for 15 min in the dark at 37°C and then incubated with 10 μl PI with gentle shaking for 10 min. Flow cytometry (FACSCanto II, Becton, Dickinson and Company) analysis was employed for detecting apoptotic events. FlowJo software v7 (FlowJo, LLC) was used to analyze the data.

### Western Blotting

Cells were lysed in cold RIPA buffer, and the protein was separated with 10% SDS–PAGE, which was then transferred to PVDF membrane (Thermo Fisher). After that, the membrane was incubated in PBS with 5% nonfat dried milk (Mengniu, Hohhot, China) for 3 h. at 4°C. Then, the membrane was incubated with primary antibodies overnight at 4°C, and then with appropriate secondary antibody (Abcam) for 1 h at 37°C. The immune complexes were detected using ECL Western Blotting Kit (Millipore).

### Luciferase Reporter Assay

The fragment of 3′UTR of MIAT containing the putative miR-34a binding site was amplified by PCR. The PCR product was subcloned into a psiCHECK-2 vector (Promega, Madison, WI, United States) immediately downstream to the luciferase gene sequence. A psiCHECK-2 construct containing 3′UTR of MIAT with a mutant seed sequence of miR-34a was also synthesized by Genepharma Co., Ltd. (Shanghai, China). All constructs were verified by DNA sequencing. The cells were plated in 96-well clusters, then cotransfected with 100 ng constructs with or without miR-34a. At 48 h after transfection, luciferase activity was detected using a dual-luciferase reporter assay system (Promega, Madison, WI, United States) and normalized to Renilla activity.

For detecting the promoter activity of miR-34a, the putative promoter region (2500 bp upstream to miR-34a sequence) was synthesized and inserted into a pGL3-basic vector (Promega, Madison, WI, United States). The Dual-Luciferase Assay Kit was used to assess luciferase activities, following manufacturer’s protocol. The PC9/R cells were plated in 96-well clusters, then cotransfected with 100 ng pGL3-basic vector or pGL3-miR-34a, together with MIAT siRNA or negative control. At 48 h after transfection, luciferase activity was detected using a dual-luciferase reporter assay system and normalized to Renilla activity.

### RNA Immunoprecipitation (RIP)

RNA Immunoprecipitation experiments were performed using the Magna RIP RNA-Binding Protein IP Kit (Millipore, Bedford, MA, United States) and the Ago2, Dnmt3a, Dnmt3b or Dnmt1 antibody (Cell Signaling, Danvers, MA, United States) according to the manufacturer’s instructions. The cells were scraped off and lysed in complete RIP lysis buffer for 30 min. Then, 100 μl of whole cell extract was incubated with RIP buffer containing magnetic beads conjugated with anti-Ago2, anti-Dnmt3a, anti-Dnmt3b or anti-Dnmt1 antibody overnight at 4°C. Normal mouse IgG (Millipore) was used as negative control. Finally, purified RNAs in the precipitates were used to determine MIAT and miR-34a expression.

### Bisulfite Genomic Sequencing PCR (BSP) and Methylation Specific PCR (MSP)

MiR-34a promoter methylation status was measured by MSP in tissues and BSP in cells. Genomic DNA was extracted using the Qiagen FFPE DNA Kit (Qiagen, Mountain View, CA, United States). Genomic DNA (1 μg per sample) was modified with bisulfite using the EZ DNA Methylation-Gold Kit (Zymo, Orange County, CA, United States) according to the manufacturer’s instructions. The PCR products were gel extracted (Qiagen) to confirm that a single band had been obtained and were then sequenced by Beijing Genomics Institute (Wuhan, China).

Methylation-specific PCR (MSP) was performed on bisulfate-treated DNA. The methylation and unmethylation primer sequences for miR-34a promotor were used as following: Methylated: forward, 5′-GGTTTTGGGTAGGCGCGTTTC-3′, reverse, 5′-TCCTCATCCCCTTCACCGCCG-3′; unmethylated: forward, 5′-TGGTTTTGGGTAGGTGTGTTTT-3′, reverse, 5′-AATCCTCATCCCCTTCACCACCA-3′. Bisulfite-modified DNA (4 μl), the methylation (3 μl) and unmethylation primer (3 μl), 2X Taq PCR Master Mix (25 μl, BioTeke, Beijing, China) and RNase-free water (17 μl) were added to achieve a final volume of 50 μl. PCR amplification conditions were as follows: 95°C for 10 min, 94°C for 30 s, annealing for 30 s, and extension at 72°C for 45 s; a total of 35 cycles; followed by a final extension at 72°C for 10 min. A total of 10 μl of the PCR product was separated using 2.5% agarose gel electrophoresis including GoldView I nuclear staining dyes (Solarbio, Beijing, China) for 30 min, and the results were photographed and analyzed.

### Tumor Xenograft in Nude Mice

Animal experiments were approved by the Ethical Committee for Animal Research of the Third Xiangya Hospital of Central South University. The cells PC/R were transfected with MIAT siRNA or both MIAT siRNA and miR-34a inhibitor for 36 h before injection. To assess tumor growth, 100 μl of the transfected PC/R cells (1 × 10^6^) was subcutaneously injected into BALB/c Nude mice (5 mice per group, 2 months-old). One day after injection, the mice were intragastrically administrated with gefitinib (100 mg/kg) once a day for 4 weeks. The tumor sizes were measured regularly and calculated using the formula: 0.5 × *L* × *W*^2^ where *L* and *W* are the long and short diameter of the tumor, respectively.

### Immunohistochemical (IHC) Staining

The parrffin-embedded tumor sections of xenografts were serially cut at 4 μm. Slides were deparaffinized and dehydrated. Slides were retrieved in citric acid buffer (pH6.0) by microwave oven. After cooling, the slices were blocked by normal goat serum, and incubated with primary antibody rabbit polyclonal anti-Ki67 antibody (1:100 dilution, Abcam) overnight at 4°C. The sections were then washed with PBS and incubated with anti-rabbit secondary antibody for 2 h at 37°C. The sections were then washed with PBS and stained by using DAB Detection Kit (Maxim, Xiamen, China). Finally, the sections were counterstained with hematoxylin. The images were obtained by using microscope (clipse Ni-E, Nikon, Japan). The quantification of positive signaling was obtained by Image-J. The data of Ki67 were acquired from eight fields/eight sections/animal and the data were averaged to produce a single value per subject.

### TUNEL Assay

The TUNEL method was used for detecting DNA breaks by using a One Step TUNEL Apoptosis Assay Kit (cat no. C1088, Beyotime, Hangzhou, China) according to the manufacturer instructions. Tissues were counterstained with 4′,6-diamidino-2-phenylindole (DAPI). Fluorescent images were acquired with a Leica DM IRB epifluorescence microscope. The quantification of positive cells was obtained by Image-J. The data of luciferase intensity were acquired from eight fields/eight sections/animal and the data were averaged to produce a single value per subject.

### Statistical Analysis

In this study, all experiments were repeated at least three times, and data are expressed as the mean ± SEM SPSS 18.0 software package (SPSS, Chicago, IL, United States) was used to perform statistical analysis. The Inhibitory concentration 50 (IC50) values were taken from the minimal experimental concentration showing 50% cell death and calculated using GraphPad Prism 7. Difference between two groups was compared by independent-samples *t* test. Difference among three or more groups was compared by One-Way ANOVA with *post hoc* Bonferroni test. The clinical association between MIAT expression and clinicopathological variables in lung cancer patients was evaluated by chi-square test. The *P* value less than 0.05 were considered statistically significant.

## Results

### High lncRNA MIAT Is a Poor Predictor for Lung Cancer Prognosis

**FIGURE 1:**
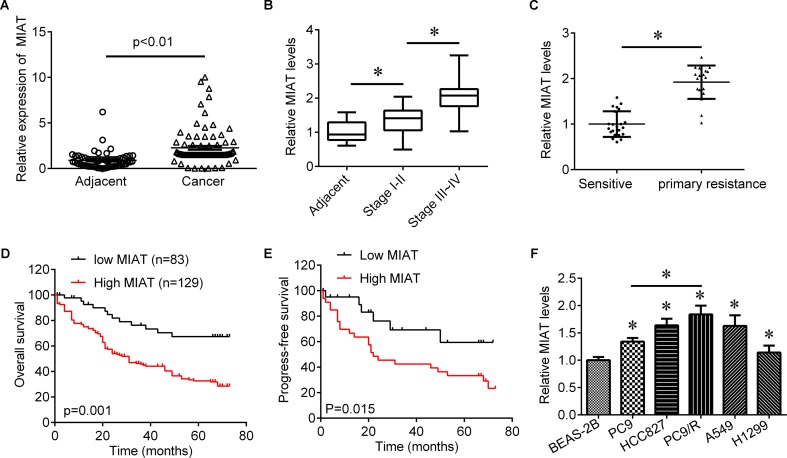
The expression of MIAT in lung cancer tissues. **(A)** qRT-PCR was used to determine the expression of MIAT in NSCLC tissues (*N* = 212) and adjacent control (*N* = 186). **(B)** The expression of MIAT in adjacent tissues and grade I+II and grade III+IV lung cancer tissues. **(C)** The expression of MIAT in patients who sensitive (*N* = 45) or primary resistant (*N* = 25) to EGFR-TKI. **(D)** Overall survival analysis in NSCLC patients with low or high MIAT expression. **(E)** Progress-free survival analysis in NSCLC patients with low or high MIAT expression. **(F)** The expression of MIAT in BEAS-2B and lung cancer cell lines. ^∗^*P* < 0.05.

To investigate role of lncRNA MIAT in lung cancer, we first evaluated the expression of lncRNA MIAT in lung cancer tissues. We found that lncRNA MIAT was significantly increased in lung cancer tissues compared with adjacent control (**Figure 1A**). In addition, the expression of lncRNA MIAT was higher in TNM stage III-IV than in TNM stage I-II (**Figure 1B**), suggesting the closed relationship of lncRNA MIAT in lung cancer development. Moreover, we divided the patients into two groups according to the median of lncRNA MIAT and found that lncRNA MIAT expression was associated with tumor size (*p* = 0.005), lymph node metastasis (*p* = 0.007), distant metastasis (*p* = 0.006) and TNM stage (*p* = 0.003), but not associated with age (*p* = 0.481), gender (*p* = 0.560) and histology type (*p* = 0.193) (**Table 1**). In addition, we investigated the factors that could predicate the prognosis of lung cancer patients by using the univariate and multivariate analyses. Univariate analysis indicated that the lncRNA MIAT level (*p* = 0.03), as well as the tumor size (*p* = 0.02), lymph node metastasis (*p* = 0.01), distant metastasis (*p* = 0.04) and TNM stage (*p* = 0.04) was significantly associated with patients’ prognosis (**Table 2**). Multivariate analysis revealed that the lncRNA MIAT level (*p* = 0.01), the tumor size (*p* = 0.04), lymph node metastasis (*p* = 0.01), distant metastasis (*p* = 0.02) and TNM stage (*p* = 0.01) were found to be independent factors for predicating the prognosis of lung cancer patients (**Table 3**). Furthermore, we found that the patients with low lncRNA MIAT have longer overall survival time and progress-free survival time than that of with high lncRNA MIAT expression (**Figures 1D,E**). And we also wanted to know the expression of lncRNA MIAT in drug resistant lung cancer tissues and cell lines. Importantly, we found that the expression of lncRNA MIAT was significantly increased in the patients who were primary resistant to EGFR-TKI (*N* = 26) compared with the patients who were sensitive to EGFR-TKI (*N* = 42) (**Figure 1C**). In line with in the tissues, the expression of lncRNA MIAT was also significantly increased in lung cancer cells compared with normal pulmonary epithelial cells BEAS-2B, and especially lncRNA MIAT was significantly increased in gefitinib-resistant PC9 (PC9/R) cells than in the parental cells (**Figure 1F**), indicating that upregulation of lncRNA MIAT may be involved in lung cancer drug resistance.

**Table 1:** Clinical association between MIAT expression and clinicopathological variables in lung cancer patients.

Variable	MIAT		χ^2^ test *p* value
	Low expression (*n* = 83)	High expression (*n* = 129)	
**Age**			
<60	39	54	0.481
≥60	44	75	
**Gender**			
Male	50	84	0.560
Female	33	45	
**Tumor size**			
<5cm	57	63	0.005
≥5cm	26	66	
**Histology type**			
Adenocarcinoma	36	44	0.193
Squamous	47	85	
**Lymph node metastasis**			
N0-1	51	54	0.007
N2-3	32	75	
**Distant metastasis**			
No	66	79	0.006
Yes	17	50	
**TNM stage**			
I-II	63	72	0.003
III-IV	20	57	

**Table 2:** Univariate analysis of prognostic factors of lung cancer.

Variable	Hazard ratio	*p* value
Age (≥60/ <60)	1.46	0.55
Gender (Male/Female)	1.03	0.82
Tumor size (≥5cm/ <5cm)	2.64	0.02
Histology type (Adenocarcinoma/Squamous)	1.08	0.68
Lymph node metastasis (N0-1/N2-3)	3.24	0.01
Distant metastasis (Yes/No)	4.21	0.02
TNM stage (III-IV/I-II)	2.25	0.04
MIAT expression (High/Low)	2.74	0.03

**Table 3:** Multivariate analysis of independent prognostic factors of lung cancer.

Variable	Hazard ratio	*p* value
Tumor size	2.36	0.04
Lymph node metastasis	2.87	0.01
Distant metastasis	3.69	0.02
TNM stage	2.74	0.02
MIAT expression	3.53	0.01

### Knockdown of lncRNA MIAT by siRNA Sensitizes Lung Cancer Cells to Gefitinib

**FIGURE 2:**
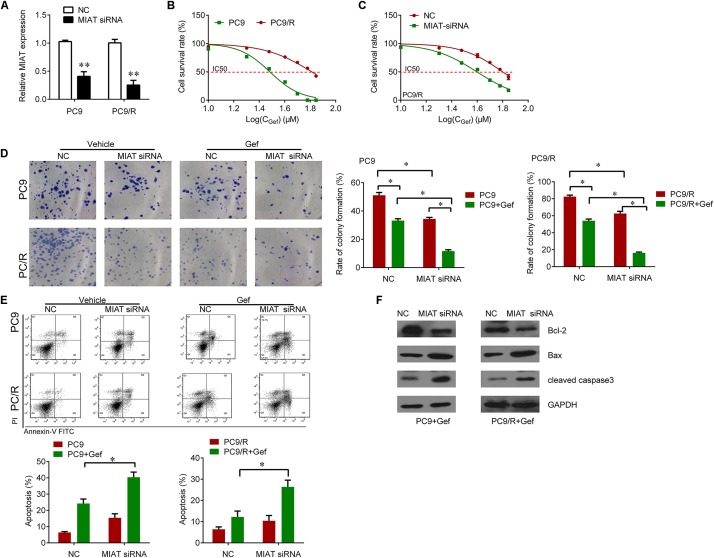
Knockdown of MIAT sensitizes PC9 and PC9/R cells to gefitinib. **(A)** qRT-PCR analysis for MIAT in PC9 and PC9/R cells after transfection with MIAT siRNA or negative control. **(B)** CCK-8 assay was used to determine the IC50 in PC9 and PC9/R cells (PC9 vs. PC9/R: 29.05 vs. 62.61 μM). **(C)** CCK-8 assay was used to determine the change of IC50 in PC9/R cells after knockdown of MIAT (siMIAT vs.NC: 38.2 vs. 60.2 μM). **(D)** Colony formation assay was used to determine the cell growth after knockdown of MIAT and gefitinib treatment. **(E)** Flow cytometry was used to measure cell apoptosis after knockdown of MIAT and gefitinib treatment. **(F)** Western blot analysis for apoptosis marker Bcl-2, Bax and cleaved caspase 3 after knockdown of MIAT and gefitinib treatment. ^∗^*P* < 0.05, ^∗∗^*P* < 0.01.

We further investigated whether knockdown of lncRNA MIAT expression could overcome the gefitinib resistance in PC9/R cells. We knocked down the expression of lncRNA MIAT in PC9 and PC9/R cells (**Figure 2A**). The IC50 of PC9/R was significantly higher than that of in PC9 cells (**Figure 2B**). Interestingly, we observed that knockdown of lncRNA MIAT significantly reduced IC50 in PC9/R cells (**Figure 2C**). Furthermore, we also found that knockdown of lncRNA MIAT enhanced the inhibitory effects of gefitinib on the inhibition of cell proliferation and promotion of cell apoptosis evaluated by significantly reduced colony formation (**Figure 2D**) and the significant increase of apoptosis rate as compared to the negative control (**Figure 2E**). We also found that knockdown of lncRNA MIAT decreased the Bcl-2 expression and increased the Bax and cleaved caspase 3 expression (**Figure 2F**). Thus, knockdown of lncRNA MIAT can overcome gefitinib resistance in lung cancer cells.

### LncRNA MIAT Mediates Hypermethylation of miR-34 Promotor

**FIGURE 3:**
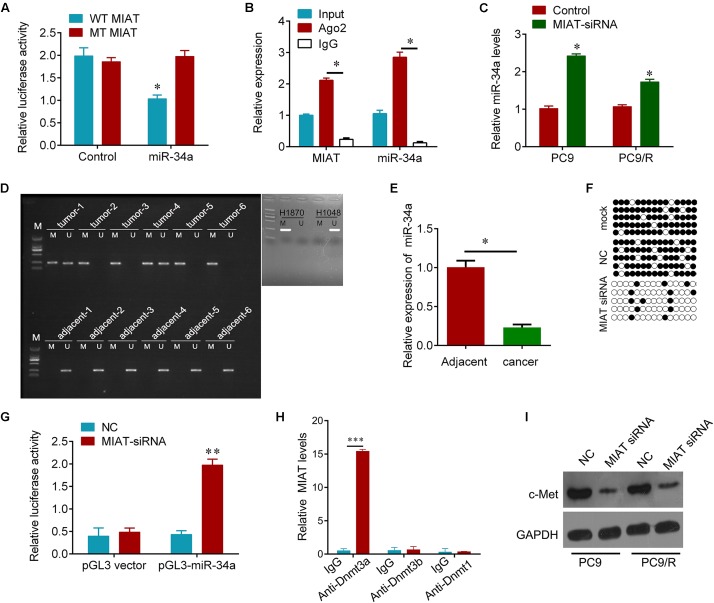
MIAT epigenetically regulates miR-34a. **(A)** The relative luciferase activities were inhibited in the PC9 cells co-transfected with wild-type MIAT 3′UTR vector and miR-34a, but not with the mutant-type vector. Firefly luciferase activity was normalized to Renilla luciferase. **(B)** Association of MIAT and miR-34a between with Ago2 in PC9 cells. Cellular lysates from PC9 cells were used for RIP with antibody against Ago2. MIAT and miR-34a expression levels were detected using qRT-PCR. **(C)** qRT-PCR analysis for miR-34a in PC9 and PC9/R cells after transfection with MIAT siRNA or negative control. **(D)** MSP test results indicated that methylation of miR-34a promoter was higher in lung cancer tissues than that of in matched adjacent tissues. H1870 cells were used as positive control and H1048 cells were used as negative control. M, methylation; U, unmethylation. **(E)** qRT-PCR analysis for miR-34a in lung cancer tissues and matched adjacent tissues in **(D)**. *N* = 6. **(F)** The BSP detection results demonstrated that methylation of miR-34a was decreased after MIAT siRNA transfection in PC9 cells. Solid circle, methylation; hollow circle, unmethylation. PCR products amplified from bisulfite-treated genomic DNA were cloned and sequenced to reveal the methylation status of individual CpG sites. Percentages of the methylated CpG sites (filled circles) among all scored sites are indicated. **(G)** Luciferase reporter analysis of luciferase activity in PC9 cells cotransfected with pGL3-miR-34a and MIAT siRNA lentivirus or an empty lentivirus. **(H)** Association of MIAT and Dnmts in PC9 cells. Cellular lysates from PC9 cells were used for RIP with antibody against Dnmt3a, Dnmt3b or Dnmt1. MIAT expression levels were detected using qRT-PCR. IgG was used as a negative control. **(I)** Western blot analysis for validated miR-34a target, c-Met after indicated treatment. GAPDH was used a loading control. ^∗^*P* < 0.05, ^∗∗^*p* < 0.01, ^∗∗∗^*p* < 0.001.

Bioinformatics analysis was used to search for the potential targeted microRNAs of lncRNA MIAT, such as MiRcode, RNAplex, catRAPID omics. Together, miR-34a was found to be a conserved target of lncRNA MIAT. To investigate if the predicted binding site of miR-34a to 3′UTR of lncRNA MIAT is responsible for this regulation, we cloned the 3′UTR of lncRNA MIAT downstream to a luciferase reporter gene (WT MIAT), its mutant version (MT MIAT) by the binding site mutagenesis was also constructed. The luciferase activity of cells co-transfected miR-34a and WT MIAT was significantly reduced compared to negative control cells. Moreover, miR-34a-mediated repression of luciferase activity was abolished by the mutant putative binding site (**Figure 3A**). The RNA-induced silencing complex (RISC) is a major mechanism of miRNAs to silence target genes, and Ago2 protein is a key constituent of RISC complex. To validate the presence of both miR-34a and lncRNA MIAT in the RISC complex, a RIP experiment was conducted using Ago2 antibody to confirm that both miR-34a and lncRNA MIAT were found in the Ago2 pellet (**Figure 3B**). We then wanted to know whether lncRNA MIAT can regulate the expression of miR-34a. We found that knockdown of lncRNA MIAT significantly increased the expression of miR-34a in PC9 and PC9/R cells (**Figure 3C**). And these regulatory effects subsequently influence the expression of targets of miR-34a. Knockdown of lncRNA MIAT significantly reduced the expression of c-Met, a validated target of miR-34a (**Figure 3I**).

We further investigated how lncRNA MIAT controls the expression of miR-34a. Promotor hypermethylation of miR-34a has been found in various cancers (Schmid et al., 2016). We here also found that the promotor of miR-34a was hyper-methylated in primary resistant lung cancer tissues, but not found in adjacent tissues; H1870 and H1048 cells were used as positive and negative control for methylation of miR-34a (Tanaka et al., 2012) (**Figure 3D**). And the expression of miR-34a was significantly downregulated in these lung cancer tissues compared with adjacent tissues (**Figure 3E**). Interestingly, we observed that knockdown of lncRNA MIAT diminished hypermethylation of miR-34a promoter (**Figure 3F**). And dual-Luciferase reporter analysis showed that knockdown of lncRNA MIAT could activate the activity of miR-34a promotor (**Figure 3G**). Moreover, RIP experiment results showed that MIAT could interact with Dnmt3a but not Dnmt3b and Dnmt1 (**Figure 3H**). These results indicate that lncRNA MIAT can epigenetically control miR-34a expression by recruiting Dnmt3a to miR-34a promotor in lung cancer cells.

### LncRNA MIAT Sensitizes Lung Cancer Cells to Gefitinib through miR-34a and PI3K/Akt/c-Met Signaling

**FIGURE 4:**
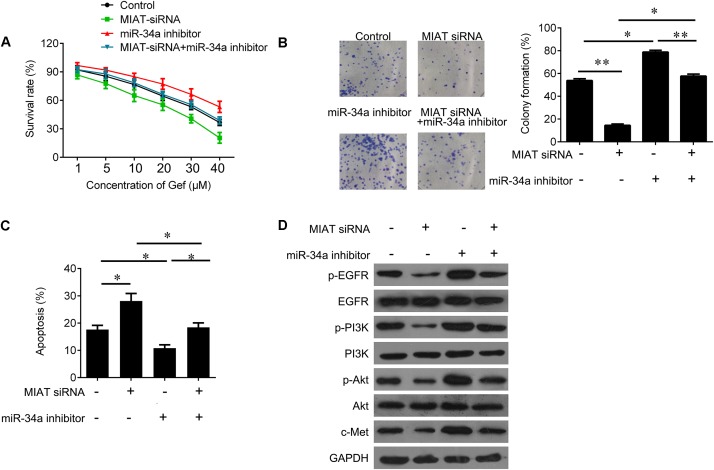
MiR-34a inhibitor reverses the inhibitory effects of MIAT downregulation on PC9 cells. **(A)** CCK-8 assay was used to determine the cell viability after miR-34a inhibitor and MIAT siRNA treatment in PC9 cells. **(B)** Colony formation assay was used to determine the cell growth after miR-34a inhibitor and MIAT siRNA treatment in PC9 cells. **(C)** Flow cytometry was used to measured cell apoptosis after miR-34a inhibitor and MIAT siRNA treatment in PC9 cells. **(D)** Western blot analysis for p-EGFR, EGFR, p-PI3K, PI3K, p-AKT, AKT and c-Met after miR-34a inhibitor and MIAT siRNA treatment in PC9 cells. ^∗^*P* < 0.05, ^∗∗^*P* < 0.01.

**FIGURE 5:**
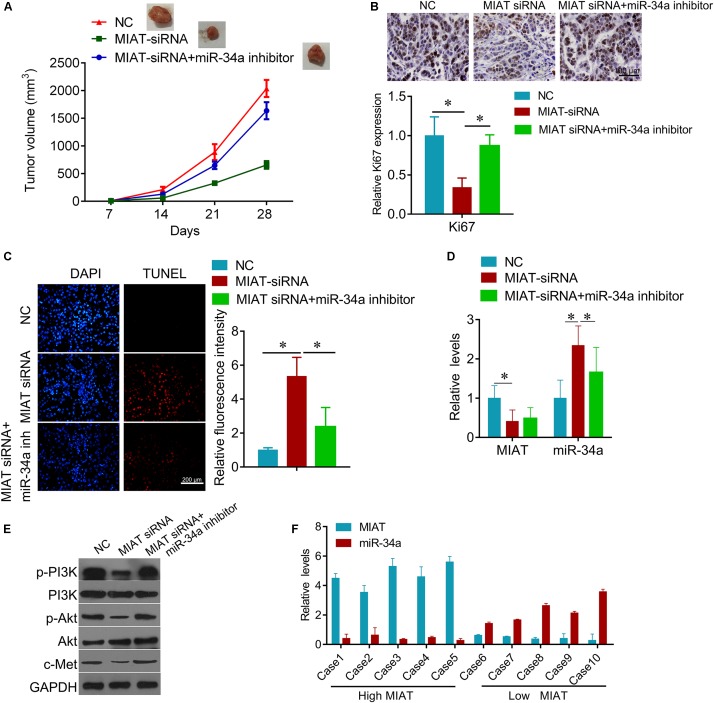
Knockdown of MIAT inhibits lung cancer growth *in vivo*. **(A)** the PC9/R cells transfected with MIAT siRNA, or together with miR-34a inhibitor were injected into nude mice. The cells transfected with empty plasmid were used as negative control. Mice were euthanized and tumors obtained from mice on day 28 after injection. **(B)** IHC was used to detect the expression of Ki67 in tumor section (upper), and quantification (lower). Scale bar, 100 μm. **(C)** TUNEL was performed to detect the apoptosis cells in tumor section (left), and the quantification of luciferase intensity (right). **(D)** qRT-PCR analysis for MIAT and miR-34a expression in xenografted tumor tissues. **(E)** Western blot analysis for c-Met and PI3K signaling proteins in xenografted tumor tissues. GAPDH was used a loading control. **(F)** qRT-PCR analysis for MIAT and miR-34a expression in human lung cancer tissues with high MIAT or low MIAT. ^∗^*P* < 0.05.

**FIGURE 6:**
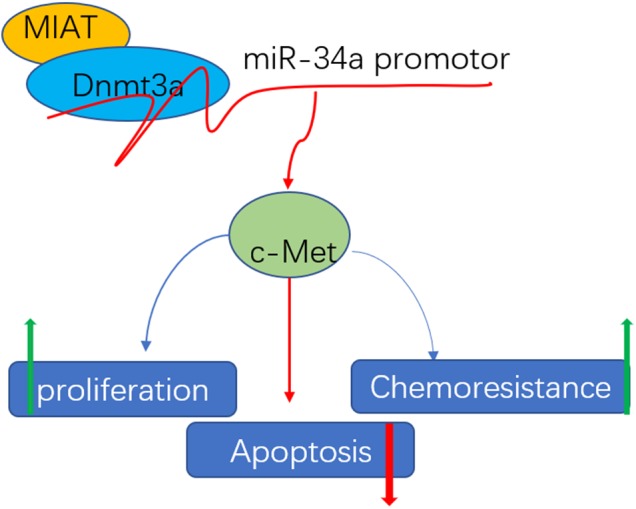
Schematic diagram of mechanism of MIAT epigenetically regulating miR-34a. MIAT interacts with Dnmt3a and recruits it on miR-34a promotor to hyper-methylate miR-34a, increasing c-Met expression, and finally conferring chemoresistance, promoting proliferation and inhibiting apoptosis.

Due to the regulatory effect between lncRNA MIAT and miR-34a, we further tested the biological role of interaction between lncRNA MIAT and miR-34a. PC9 cells were transfected with MIAT siRNA or miR-34a inhibitor alone, or both combined. We found that down-regulated miR-34a largely attenuated MIAT siRNA-mediated inhibition of cell survival (**Figure 4A**) and clonogenic ability (**Figure 4B**), and induction of cell apoptosis (**Figure 4C**). In addition, we found that knockdown of lncRNA MIAT could inactivate PI3K/Akt/c-Met signaling, whereas miR-34a inhibitor reversed this inactivation of PI3K/Akt/c-Met signaling (**Figure 4D**). Moreover, we also confirmed these results *in vivo*. Compared with NC control group, knockdown of lncRNA MIAT significantly inhibited tumor growth (**Figure 5A**). And knockdown of lncRNA MIAT inhibited the Ki67 expression and increased the TUNEL positive cells (**Figures 5B,C**). However, these inhibitory effects were reversed by miR-34a inhibitor (**Figure 5**). And we also confirmed the regulatory effects of MIAT on PI3K/Akt/c-Met signaling in xenografted tissues (**Figures 5D,E**). In addition, qRT-PCR performed in human lung cancer tissues with high (*n* = 5) or low (*n* = 5) MIAT expression showed that miR-34a expression is negatively correlated with MIAT expression (**Figure 5F**). As shown in schematic diagram of mechanism of MIAT epigenetically regulating miR-34a (**Figure 6**). It is possible that MIAT interacts with Dnmt3a and recruits it on miR-34a promotor to hyper-methylate miR-34a, reducing miR-34a expression and subsequent increasing c-Met expression. The results indicate that lncRNA MIAT enhances gefitinib resistance in lung cancer cells via targeting miR-34a.

## Discussion

In this study, we found that lncRNA MIAT was upregulated in lung cancer tissues compared with adjacent tissues, and the patients with low lncRNA MIAT had longer overall survival time and progression-free survival time than patients with high lncRNA MIAT expression. LncRNA MIAT may be an independent factor for predicating the prognosis of lung cancer patients. Increasing evidence indicates lncRNAs can be prognostic factors for lung cancer patients. Qu CH et al showed that higher expression of linc-ROR predicted significantly shorter 5-year overall survival and disease-free survival (DFS) in patients with NSCLC (Qu C.H. et al., 2017). LncRNA HOTTIP was overexpressed in small cell lung cancer tissues, and its expression was correlated with the clinical stage and the shorter survival time of small cell lung cancer patients (Sun et al., 2017). Serum lncRNA AFAP1-AS1 could be used as molecular marker for distinguishing non-small cell lung cancer patients from healthy people, and high serum lncRNA AFAP1-AS1 expression levels correlated with distant metastasis, lymph node metastasis, poor clinical stage, and larger tumor size (Li W. et al., 2017). Our results in this study population showed that the 5-year overall survival was about 25% in MIAT-High group, and about 60% in MIAT-Low group. The cancer statistics in China predicted that 36.9% of cancer patients in China will survive at least 5 years after diagnosis; and the lowest survival rates were found in Southwest China (24.9%), with Central China showing the highest rate (41.0%) (Chen W. et al., 2016). And the lung cancer 5 year-survival rate in United States from 2002 to 2008 was about 30% (Siegel et al., 2013; Torre et al., 2015). Thus, the 5-year overall survival in MIAT-High group (25%) in our study population was significantly lower, and the survival in MIAT-Low group was higher than the historical survival rate. However, we cannot exclude the difference in race and region. The subjects in this study mainly come from Hunan province (located on Central China) with relatively higher survival rate in China. Thus, we concluded that the expression of MIAT can be used to predict the outcome of patients with lung cancer.

Previous studies demonstrated that MIAT was highly expressed in cardiomyocytes, and involved in MI (Qu X. et al., 2017), diabetic cardiomyopathy (Zhou et al., 2017), ischemic stroke (Zhu et al., 2017), angiogenesis (Yan et al., 2015; Jiang et al., 2016). Recently, MIAT was found to be an oncogenic lncRNA in various cancers. Crea et al. (2016) found that LncRNA MIAT was selectively upregulated in neuroendocrine prostate cancer and might interact with Polycomb genes. Knockdown MIAT inhibited breast cancer cell proliferation, promoted apoptosis and decreased migration and invasion by acting as a competing endogenous RNA to regulate the expression of dual specificity phosphatase 7 by taking up miR-155-5p (Luan et al., 2017). Upregulation of MIAT was also related to the proliferation and invasion of hepatocellular carcinoma and gastric cancer by releasing miR-214 and miR-29a, respectively (Huang et al., 2017; Li Y. et al., 2017). By RNA-seq data and miRNA-seq data of lung adenocarcinomas, Li et al. (2016) identified three differentially expressed lncRNAs, including MIAT, were associated with clinical features involved in MAPK signaling pathways. And Zhang HY demonstrated that knockdown of MIAT significantly downregulated the expression of the zinc finger E-box binding homeobox 1 (ZEB1) through suppressing miR-150 to inhibit cell invasion of NSCLC cells (Zhang et al., 2017). Importantly, we here report that the expression of MIAT was significantly increased in patients who were primary resistant to EGFR-TKIs compared with the sensitive patients. And knockdown of MIAT by siRNA could sensitize lung cancer cells to gefitinib.

Interestingly, we found that MIAT interacted with miR-34a, and epigenetically and negatively regulated miR-34a expression by hypermethylating its promotor to control c-Met expression. Knockdown of MIAT also enhanced gefitinib cytotoxicity through inactivating PI3K/AKT signaling, an important pathway to promote cancer cell proliferation (De Marco et al., 2017). MiR-34a is an important tumor suppressor whose expression is epigenetically silenced in various human cancers (Saito et al., 2015; Adams et al., 2016). And hypermethylation of miR-34a promotor resulted in miR-34a silence in prostate cancer (Liao H. et al., 2016), ovarian cancer (Schmid et al., 2016), cholangiocarcinoma (Kwon et al., 2017), laryngeal squamous cell carcinoma (Shen et al., 2016), and lung cancer (Kim et al., 2017). Zhou et al. (2014) found that miR-34a rescued hepatocyte growth factor-induced gefitinib resistance in EGFR mutant NSCLC cells by inhibiting activity of the PI3K/Akt pathway. Zhao et al. (2017) also demonstrated that combined miR-34a mimics and EGFR-TKIs synergistically sensitized both EGFR wild-type and mutant NSCLC cells. The hypermethylation of miR-34a promotor was mediated by several DNA methyltransferases (Dnmts), including Dnmt1, Dnmt2, and Dnmt3a (Peng et al., 2015, 2017; Wang et al., 2016; Lewinska et al., 2018). Here, we show MIAT binds to the miR-34a promotor, and recruits Dnmt3a to methylate the promotor, leading to silencing of miR-34a expression.

## Conclusion

Our findings demonstrate that silencing of MIAT sensitizes lung cancer cells to gefitinib by epigenetically miR-34a, and MIAT may be a useful prognostic marker and therapeutic target for lung cancer patients.

## Author Contributions

The study and experiments were conceived and designed by JL and RG. Patients were selected by: YF and CL. The experiments were performed by YL, LL, and CL. Analyzed and discussed the data and discussed the written manuscript: all authors. The manuscript was written by YF, CL, and RG. The figures and tables were constructed by YF, YL, and CL.

## Conflict of Interest Statement

The authors declare that the research was conducted in the absence of any commercial or financial relationships that could be construed as a potential conflict of interest.
